# Hemodynamic exercise responses with a continuous-flow left ventricular assist device: Comparison of patients’ response and cardiorespiratory simulations

**DOI:** 10.1371/journal.pone.0229688

**Published:** 2020-03-18

**Authors:** Christoph Gross, Libera Fresiello, Thomas Schlöglhofer, Kamen Dimitrov, Christiane Marko, Martin Maw, Bart Meyns, Dominik Wiedemann, Daniel Zimpfer, Heinrich Schima, Francesco Moscato

**Affiliations:** 1 Center for Medical Physics and Biomedical Engineering, Medical University of Vienna, Vienna, Austria; 2 Ludwig Boltzmann Institute for Cardiovascular Research, Vienna, Austria; 3 Department of Cardiac Surgery, Katholieke Universiteit Leuven, Leuven, Belgium; 4 Institute of Clinical Physiology, National Research Council, Pisa, Italy; 5 Department of Cardiac Surgery, Medical University of Vienna, Vienna, Austria; 6 PVA Center for Ambulatory Rehabilitation Vienna, Vienna, Austria; Thomas Jefferson University, UNITED STATES

## Abstract

**Background:**

Left ventricular assist devices (LVADs) are an established treatment for end stage heart failure patients. As LVADs do not currently respond to exercise demands, attention is also directed towards improvements in exercise capacity and resulting quality of life. The aim of this study was to explore hemodynamic responses observed during maximal exercise tests to infer underlying patient status and therefore investigate possible diagnostics from LVAD derived data and advance the development of physiologically adaptive LVAD controllers.

**Methods:**

High resolution continuous LVAD flow waveforms were recorded from 14 LVAD patients and evaluated at rest and during maximum bicycle exercise tests (n = 24). Responses to exercise were analyzed in terms of an increase (↑) or decrease (↓) in minimum (Q_MIN_), mean (Q_MEAN_), maximum flow (Q_MAX_) and flow pulsatility (Q_P2P_). To interpret clinical data, a cardiorespiratory numerical simulator was used that reproduced patients’ hemodynamics at rest and exercise. Different cardiovascular scenarios including chronotropic and inotropic responses, peripheral vasodilation, and aortic valve pathologies were simulated systematically and compared to the patients’ responses.

**Results:**

Different patients’ responses to exercise were observed. The most common response was a positive change of ΔQ_MIN_↑ and ΔQ_P2P_↑ from rest to exercise (70% of exercise tests). Two responses, which were never reported in patients so far, were distinguished by Q_MIN_↑ and Q_P2P_↓ (observed in 17%) and by Q_MIN_↓ and Q_P2P_↑ (observed in 13%). The simulations indicated that the Q_P2P_↓ can result from a reduced left ventricular contractility and that the Q_MIN_↓ can occur with a better left ventricular contractility and/or aortic insufficiency.

**Conclusion:**

LVAD flow waveforms determine a patients’ hemodynamic “fingerprint” from rest to exercise. Different waveform responses to exercise, including previously unobserved ones, were reported. The simulations indicated the left ventricular contractility as a major determinant for the different responses, thus improving patient stratification to identify how patient groups would benefit from exercise-responsive LVAD control.

## Introduction

Left ventricular assist devices (LVADs) have become an established therapy to manage end-stage heart failure [1]. Patients receive an LVAD with different treatment intentions: as a bridge to cardiac transplant, as a bridge to further treatment decision, as bridge to cardiac recovery, and even for lifetime implantation (so-called destination therapy). This results in a patient population with a broad spectrum of demographic parameters and comorbidities with individual pathophysiological conditions. Patients typically benefit of hemodynamic normalization at rest after LVAD implantation.

Despite increasing implantation rates over the last decade with improvements in patient outcomes and acceptable durability of currently used LVADs [1], exercise capacity remain substantially lower compared to gender and age predicted values [2]. Exercise capacity, often referred to by measurements of oxygen uptake at peak exercise, involves mechanisms at multiorgan levels with a prominent component related to cardiac function [2,3]. Exercise response in LVAD patients may reflect underlying differences in cardiac and peripheral conditions, as well as in the interaction between the assisted left ventricle and the LVAD, strongly tied to the overall cardiac output. This study is an attempt to characterize these patients’ cardiac and peripheral conditions at the level of the pump flow waveform and hypothesize their determinants.

Currently used LVADs operate at a fixed pump speed with different resulting supporting levels. When the cardiac demand is solely delivered by the pump one speaks of full-support. With a partial ventricular support by the LVAD, an additional volume of blood is ejected through the aortic valve in parallel to the output provided by the LVAD. Improvement in cardiac output with exercise results with fixed LVAD operation from the adaptations of the patient’s cardiovascular system, rather than from an increase in pump output [4], thus indicating possible improvements by exercise-responsive LVADs. However, a proper characterization of patient residual adaptation mechanisms to exercise is important for the design of exercise-responsive LVADs. High-resolution LVAD flow waveforms have proven to be a valuable source of information for the detection of patient’s hemodynamic status [5]: Aortic valve (AV) opening [6,7], heartrate [8], suction events [9], contractility and relaxation parameters [10,11]. This LVAD-based diagnostics can be therefore performed systematically during exercise tests, thus revealing the unobserved information of this complex interaction between heart and LVAD. The aim of this study was to evaluate, for the first time ever, patients’ hemodynamic LVAD flow waveform responses during exercise and compare the results to the responses reproduced with systematic cardiorespiratory numerical simulations (performed independent from responses observed in patients). This should help to design physiologically adaptive controls which take into account different types of exercise as well as individual patient conditions.

## Methods

This work consists of a clinical study conducted on 14 patients that underwent a total of 24 maximal bicycle exercise tests while high resolution LVAD data were recorded. The LVAD data of these patients collected from rest to exercise were analyzed and compared to the LVAD data resulting from a cardiorespiratory simulator. The cardiorespiratory simulator reproduced hemodynamics during exercise for the average LVAD patient from literature with additional single cardiovascular parameter changes described in detail below.

### Patient analysis

Patient data was collected within a prospective observational study approved by the Institutional Review Board of the Medical University Vienna (EK-243/2011, ClinicalTrial.gov identifier: NCT01981642). High resolution LVAD data (motor current and impeller rotational speed) of patients implanted with an HVAD (Medtronic Inc., Minneapolis, MN, USA) during maximum bicycle exercise tests were recorded continuously with a sampling frequency of 50 Hz. Pharmacologic treatment of patients was in accordance with the guidelines of the European Society of Cardiology [12] and included beta-blocker, ACE-inhibitor, diuretica, calcium-channel blockers and Angiotensin II type I receptor blockers. Mean arterial blood pressure levels from 70–85 mmHG in these patients were desired. Echocardiographic examination of the right ventricular function was assessed according to the recommendations of the American college of cardiology [13]. The classification of the right ventricular function was made based on global visual assessment [13,14] and staged as normal or, in case of dysfunction as mild, moderate or severe dysfunction.

Patients’ exercise tests were performed on a bicycle ergometer and included cardiopulmonary exercise tests (upright position) and bicycle stress-echocardiography (semi-recumbent position). The incremental increase in workload was determined by the clinician on a patient-individual basis, according to previous results of maximum or sub-maximum physical capacity tests. Bicycle ergometer tests were performed until subjectively perceived maximum physical capacity symptoms developed. Additional information concerning the performed tests is provided in reference [15].

**Fig 1:**
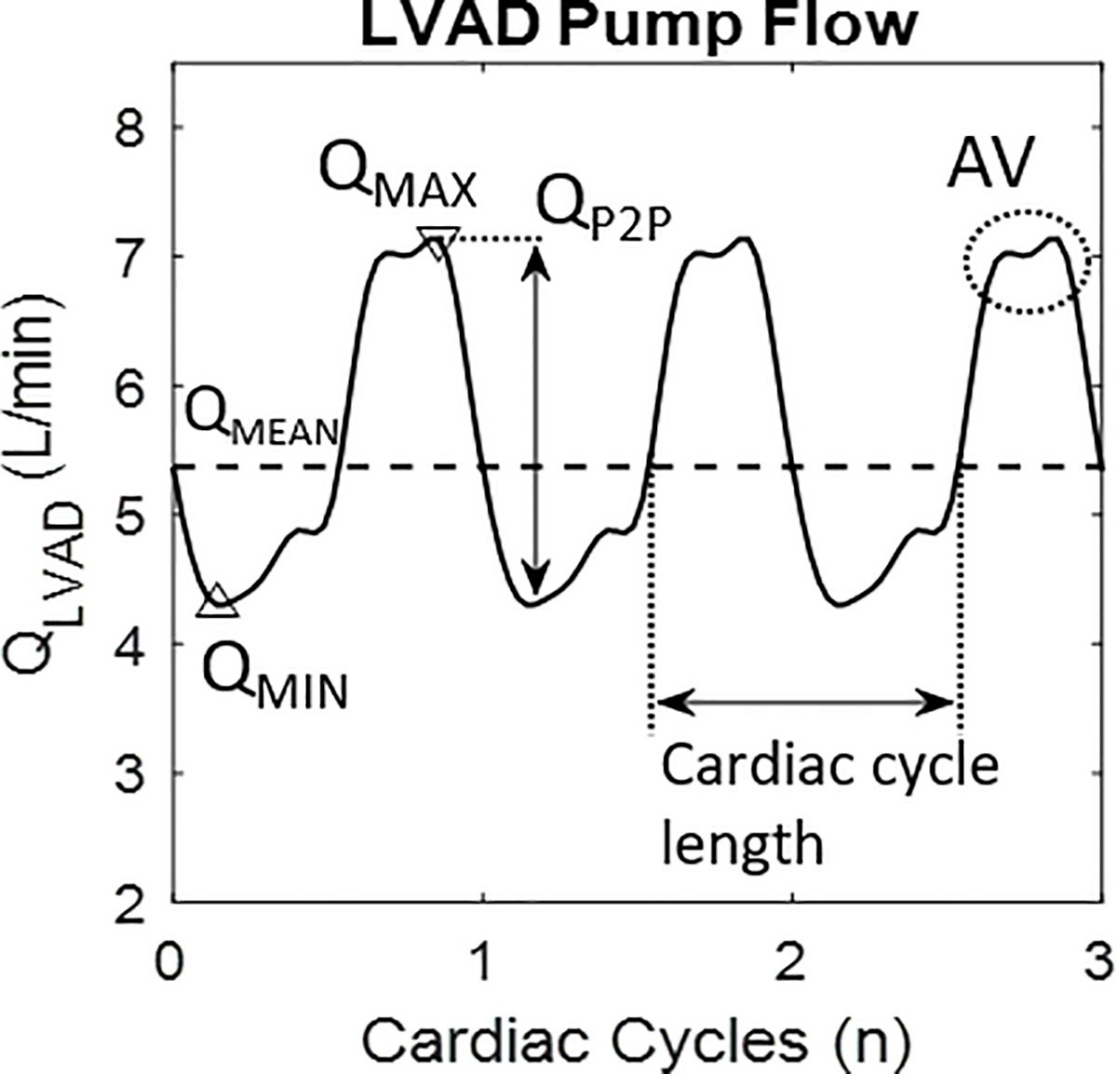
Analyzed LVAD waveform parameters. LVAD pump flow features analyzed to characterize patients’ response to exercise. For description of symbols please refer to the text.

Pump flow was estimated from the recorded LVAD motor current and impeller rotational speed [16] and heartrate (HR) was derived subsequently using a previously developed method [8]. For each cardiac cycle the following hemodynamic features were calculated (see Fig 1): pump flow minimum (Q_MIN_), mean (Q_MEAN_), maximum (Q_MAX_) values, flow pulsatility (Q_P2P_) and aortic valve (AV) opening [6,17]. LVAD waveform responses were classified by the changes of the parameters Q_MIN_, Q_MEAN_, Q_MAX_ and Q_P2P_. The symbols ↓ and ↑ will be used to indicate negative (<0 L/min) and “positive” (≥0 L/min) parameter changes from baseline to peak exercise.

### Statistical analysis

Data was processed and analyzed with MATLAB (TheMathworks Inc., Natick, MA, USA). Metric variables were tested for normality with the Shapiro-Wilk test for small sample sizes and are reported as mean ± standard deviation (SD) for normally distributed data. Non-normally distributed data is reported as median with 25^th^ and 75^th^ percentile (quartile 1 & 3). Statistical significance for responses in LVAD parameters was calculated with the two sample t-test for the normal distributed metric data and with Fisher’s exact test for nominal variables. Statistical significance was set at p<0.05.

### Exercise simulations

**Fig 2:**
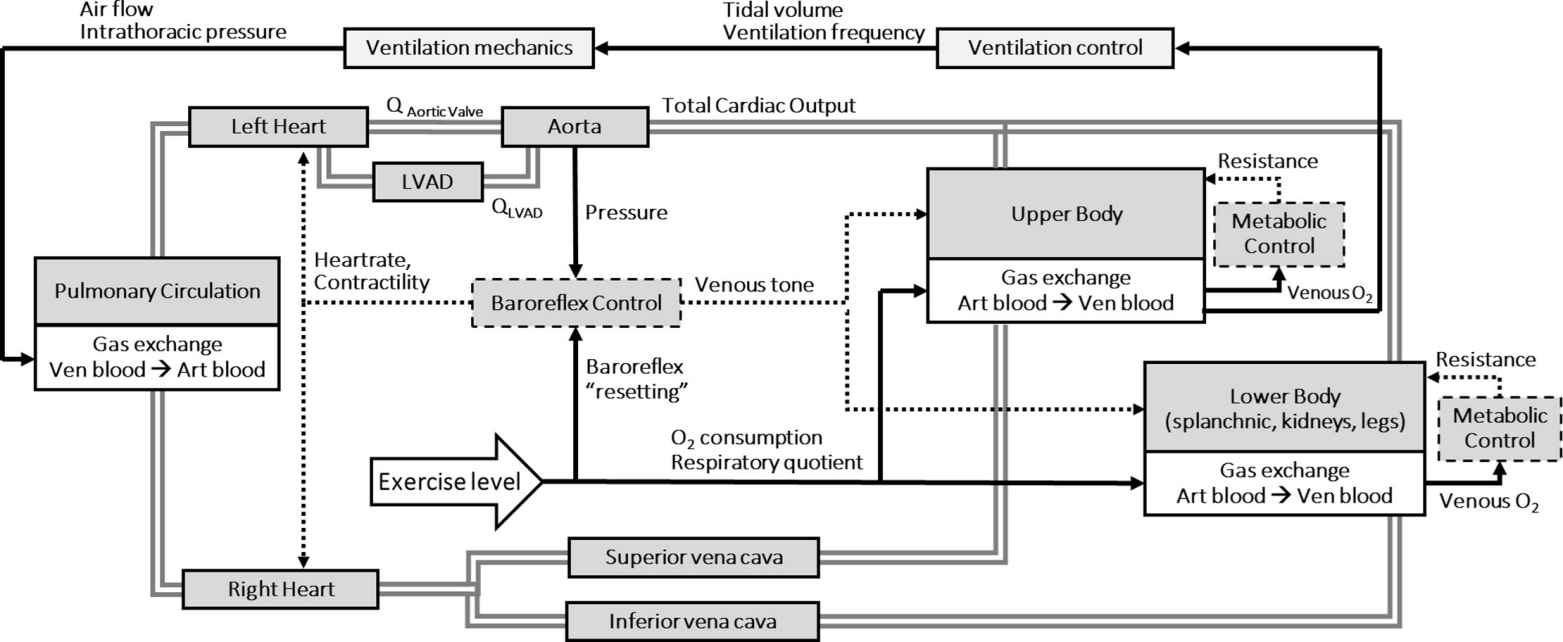
Cardiorespiratory simulator. Schematic diagram of the cardiovascular compartments and biological feedback control loops (arrows) of the cardiorespiratory simulator.

To evaluate possible hemodynamic mechanisms responsible for responses in LVAD parameters to exercise, a computational simulator was used and a sensitivity analysis for systematic single parameter changes performed. The simulator was developed at the Cardiac Surgery Department of the Katholieke Universiteit Leuven in LabVIEW (National Instruments Austin, TX, USA) (see Fig 2). The simulator included a lumped parameters model representing the cardiovascular and respiratory systems combined together and specifically adapted to reproduce heart failure conditions [18,19]. The cardiovascular system included a time varying elastance model for the representation of the atria and ventricles. The circulation is split into different circulatory compartments (ascending and descending aorta, upper body, kidneys, splanchnic circulation, left and right leg, superior and inferior vena cava and pulmonary circulation), in which the metabolic state of oxygen consumption and production of carbon dioxide can be simulated depending on physical exertion.

Specific physiological feedback mechanisms were implemented to reproduce the cardiorespiratory changes observed from rest to exercise. Autonomic control regulated sympathetic nerve overstimulation and vagal withdrawal resulting in vasoconstriction of the systemic circulation and positive inotropic and chronotropic responses of the heart during exercise. Metabolic control regulated the vasodilation in the peripheral tissues, if hypoxia was detected. Concerning ventilation, a specific control was implemented that regulates tidal volume and ventilation frequency according to the oxygen and carbon dioxide partial pressure sensed in the upper body.

The model of the LVAD was implemented according to the in-vitro measurements with a HVAD pump reported in [16] and connected between the left ventricle and the aorta (see Fig 2) [20–22].

The average hemodynamic condition of LVAD patients at rest were simulated with a LVAD flowrate of 4.8 L/min (full support, thereby equaling the cardiac output) at a LVAD speed of 2700rpm as described in [23–27]. Then, a bicycle exercise with an intensity of 80 watts was simulated, corresponding to an oxygen uptake of 15.2 ml/min/kg, a typical maximum value reported in LVAD patients at peak exercise. The simulation evolved in order to attain the exercise hemodynamic condition, with similar hemodynamic adaptations as reported by previous clinical studies [23–27]. This set of simulation parameters, that reproduce an average LVAD patient, are referred to as baseline (BL_REST_ and BL, at rest and during exercise respectively). In addition to that, other simulation runs were performed to mimic different LVAD patient profiles. For this purpose, one cardiovascular parameter at a time was decoupled from feedback control mechanisms and manually changed instead. See Table 1 for a list of these parameters and their changes.

**Table 1:** Cardiovascular simulation parameters.

	Rest		Exercise	
Simulation Label	BL_Rest_	AI_Rest_	Baseline/-20%/+20%	Simulation Label
Heart rate	75		113/90/136	BL/HR-/HR+
Left ventricular contractility (mmHg/cm^3^)	0.52		0.65/0.52/0.78	BL/EmaxL-/EmaxL+
Right ventricular Contractility (mmHg/cm^3^)	0.36		0.46/0.37/0.55	BL/EmaxR-/EmaxR+
Total peripheral resistance (mmHg*s/cm^3^)	0.92		0.47/0.37/0.56	BL/TPR-/TPR+
Aortic valve insufficiency	none	severe	none/ severe	BL/AI
Aortic valve stenosis	none	none	none/severe	BL/AS

Cardiovascular parameters at rest without and with aortic insufficiency (AI) and during exercise simulations with the labels: Baseline (BL), left ventricular contractility (EmaxL), right ventricular contractility (EmaxR), total peripheral resistance (TPR), aortic valve insufficiency (AI) and aortic valve stenosis (AS).

Heart rate (HR), left/right ventricular contractility (EmaxL/EmaxR) and total peripheral resistance (TPR) were increased and decreased by 20% compared to BL simulation at exercise. This permitted to mimic a better/poorer chronotropic, inotropic or peripheral circulatory response to exercise. Valvular pathologies, as observed in some LVAD patients, were also simulated such as aortic valve insufficiency (AI) and aortic valve stenosis (AS) [28,29]. The simulated cardiovascular parameters mentioned beforehand are reported in Table 1.

**Fig 3:**
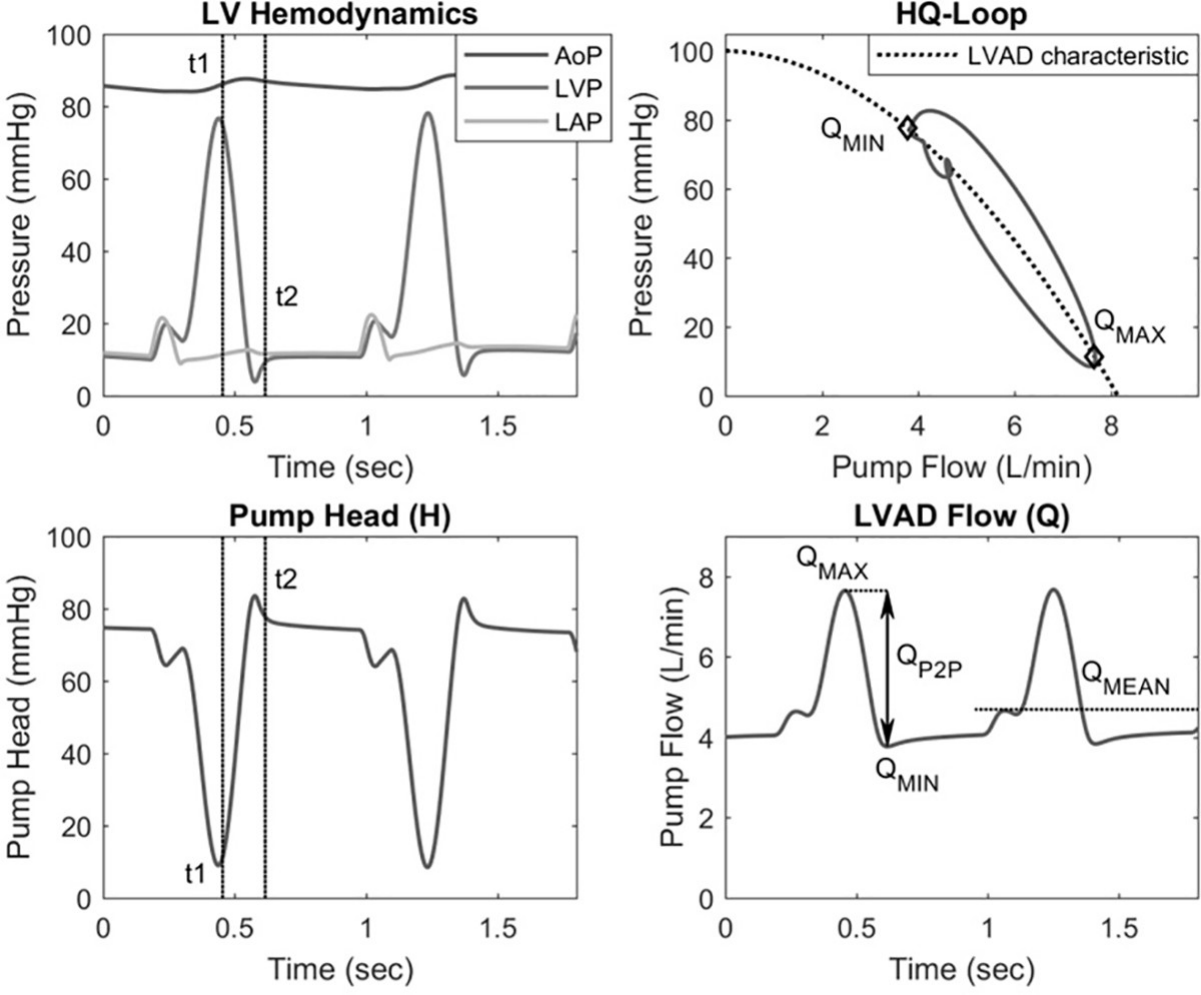
Simulated LVAD hemodynamics. Left heart pressures, pump head pressure (H) and corresponding LVAD pump flow with the analyzed flow waveform parameters from simulation. Hemodynamics corresponding to the time points at Q_MAX_ and Q_MIN_ are depicted with t1 and t2, respectively. Furthermore resulting pump flows from pump head (H = AoP-LVP) and the LVAD’s characteristic are shown as HQ-Loop (top right).

A comprehensive overview of the cardiovascular parameter profile for the baseline simulation and the parameter ranges of the performed simulations during exercise are shown in Table 1. The LVAD flow, resulting from these different simulations, was analyzed with the same signal processing routines used to analyze the clinical data (see Fig 3).

## Results

### Patient data

**Fig 4:**
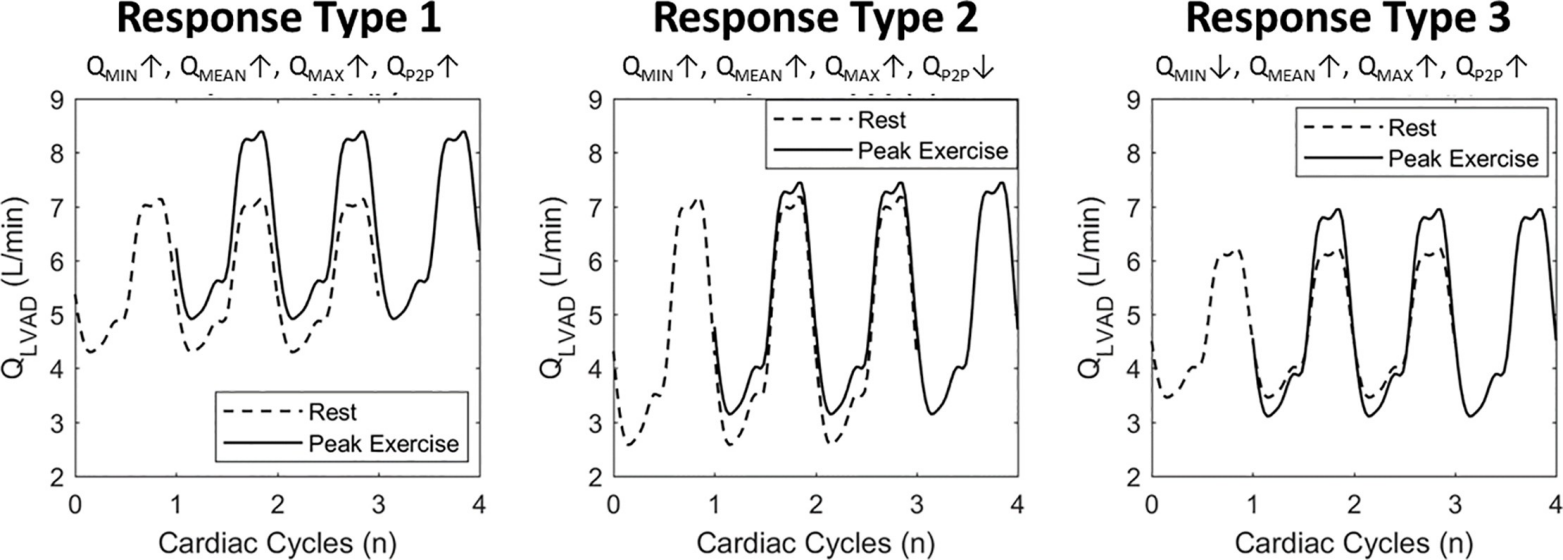
Patients’ average LVAD waveform responses. Overview of the three average responses from patients’ LVAD pump flow at rest and during exercise. Parameter values for the responses are depicted in Table 3.

A total of 24 bicycle exercise stress tests, consisting of 18 cardiopulmonary exercise tests and 6 bicycle stress-echocardiography, were analyzed from 14 patients implanted with an LVAD. Demographic, clinical and exercise data are reported in Table 2. For all bicycle exercise tests the response in parameters ΔQ_MEAN_ and ΔQ_MAX_ was always positive (↑) whereas different responses in ΔQ_MIN_ and ΔQ_P2P_ occurred. Consequently, three different types of LVAD waveform responses were identified based on the changes of ΔQ_MIN_ and ΔQ_P2P_. Response type 1 is characterized by ΔQ_MIN_↑ and ΔQ_P2P_↑ and was found in 70% of all exercise tests. This response occurred in all exercise tests of patients with the AV remaining closed at peak exercise and in 60% of all tests with the AV open at peak exercise. Response type 2 is classified by ΔQ_MIN_↑ and ΔQ_P2P_↓ and was found in 17% of all tests. Response type 3 is characterized by ΔQ_MIN_↓ and ΔQ_P2P_↑ and was found in 13% of all tests. Response type 2 and 3 were only found in tests with the patients’ AV open at peak exercise. The statistics for the subgroups of the three identified responses is provided in Table 3. Additionally examples of the changes in LVAD waveforms from rest to exercise in the three responses are shown in Fig 4. Individual LVAD parameter responses for each bicycle exercise test can be observed in the online available data of this study.

**Table 2:** Patient demographics.

	n (%) or Mean ± StD or Median (Quartile 1 & 3)
Patients	14
Gender (male/female)	12/2 (86%/14%)
Age (years)	58.9 ± 10.8
BMI (kg/m^2^)	27.2 ± 4.8
Intermacs level: n	1: 4 (29%)
	2: 1 (7%)
	3: 5 (35%)
	4: 4 (29%)
Etiology: CMP (isc. / non-isc.)	7/7 (50%/50%)
LVAD indication	BTT: 6 (43%)
	BTC: 3 (21%)BTR: 1 (7%)
	DT: 4 (29%)
**Co-morbidities**	
Diabetes mellitus	5 (36%)
Pulmonary hypertension	4 (29%)
Arterial hypertension	7 (50%)
Atrial fibrillation	3 (21%)
ICD	11 (79%)
Renal insufficiency	3 (21%)
COPD	3 (21%)
**Medication**	
Angiotensin-converting enzyme inhibitors	10 (71%)
Beta-blockers	12 (86%)
Diuretics	9 (64%)
Calcium-channel blockers	4 (29%)
Angiotensin II type I receptor blockers	2 (14%)
**Echocardiography**	
Right ventricular function	Normal: 1 (7%)
	Dysfunction: 13 (93%)
	mild: 6 (43%)
	moderate: 6 (43%)
	severe: 1 (7%)
**Bicycle Exercise Tests (n = 24)**	
VAD support (post operative days)	80 (59 & 431)
VAD speed (rpm)	2900±200
CPET / Bicycle stress-echocardiography	18/6 (75%/25%)
Peak workload per bodyweight (W/kg)	0.49 (0.36 & 0.64)
Exercise duration (min)	7.7±1.8
pVO2 from CPETs (ml/kg/min)	9.5±2.2
Respiratory exchange ratio from CPETs	1.1±0.1

Demographics, medications, right ventricular function assessed by echocardiography and type and measures of exercise tests of the patients studied to analyze response in LVAD parameters during maximum bicycle exercise. BMI = body mass index, Intermacs = Interagency Registry for Mechanically Assisted Circulatory Support, CMP = cardiomyopathy, isc = ischemic, CPET: Cardiopulmonary exercise test.

**Table 3:** Patients’ LVAD waveform responses.

	Response Type 1 (ΔQ_MIN_↑ and ΔQ_P2P_↑)	Response Type 2 (ΔQ_MIN_↑ and ΔQ_P2P_↓)	Response Type 3 (ΔQ_MIN_↓ and ΔQ_P2P_↑)
	Baseline → peak Exercise (Delta)	Baseline → peak Exercise (Delta)	Baseline → peak Exercise (Delta)
**Q**_**MAX**_ **(L/min)**	7.1±1.0 → 8.4±0.9 (Δ = +1.3±0.9, p = 0.001)	7.2±1.1 → 7.4±1.2 (Δ = +0.3±0.1, p = 0.8)	6.2±0.5 → 6.9±0.7 (Δ = +0.7±0.6, p = 0.2)
**Q**_**MEAN**_ **(L/min)**	5.4±0.8 → 6.4±0.8 (Δ = +1.0±0.6, p = 0.001)	4.6±0.9 → 5.2±1.1 (Δ = +0.6±0.3, p = 0.4)	4.6±0.5 → 5.0±0.5 (Δ = +0.5±0.3, p = 0.3)
**Q**_**MIN**_ **(L/min)**	4.3±1.0 → 4.9±1.0 (Δ = +0.6±0.4, p = 0.09)	2.6±1.4 → 3.1±1.3 (Δ = +0.6±0.3, p = 0.6)	3.5±0.5 → 3.1±0.6 (Δ = -0.3±0.2, p = 0.5)
**Q**_**P2P**_ **(L/min)**	2.8±1.1 → 3.5±0.9 (Δ = +0.6±0.6, p = 0.07)	4.6±1.3 → 4.3±1.2 (Δ = -0.3±0.2, p = 0.7)	2.8±0.1 → 3.8±0.7 (Δ = +1.1±0.8, p = 0.06)
**Heartrate (bpm)**	74±13 → 90±21 (Δ = +17±15, p = 0.01)	71±6 → 91±13 (Δ = +19.8±8.3, p = 0.03)	75±10 → 107±11 (Δ = 31±16, p = 0.02)
**LVAD Speed (rpm)**	2942±222	2674±206	2766±116
**Aortic Valve open/closed (n)**	2/15 → 10/7 (p = 0.01)	3/1 → 4/0 (p = 1)	1/2 → 3/0 (p = 0.4)
**Bicycle Exercise Tests (n)/Patients (n)**	17/10	4/4	3/2
**CPET (n) / Bicycle Stress- Echocardiography (n)**	14/3	2/2	2/1
**Workload (W/kg)**	0.4 (0.3 & 0.7)	0.4 ±0.2	0.7±0.1
**Duration (min)**	8.0 ±1.8	6.4 ±1.6	7.7±2.2
**pVO2 from CPET**	8.7 (7.5 & 9.6)	{11.3 & 9.1}	{10.2 & 11.4}
**RV dysfunction (grade)**	mild: n = 4 Patients moderate: n = 5 Patients no dysfunction (normal): n = 1 Patient	moderate: n = 2 Patients severe: n = 1 Patient no dysfunction (normal): n = 1 Patient	mild: n = 2 Patients

Statistical analysis of patient responses in LVAD derived parameters during maximum bicycle exercise testing. (CPET: Cardiopulmonary Exercise Test, RV: right ventricular, {…}: individual values reported due to CPETs n = 2)

Five of the 14 patients underwent multiple bicycle exercise tests. Two of these five patients showed intra-individual differences in responses, one patient due to different AV status at rest (Patient Nr. 11 in S1 Table) and the other due to the occurrence of suction (Patient Nr. 2 in S1 Table).

### Simulation data

**Fig 5:**
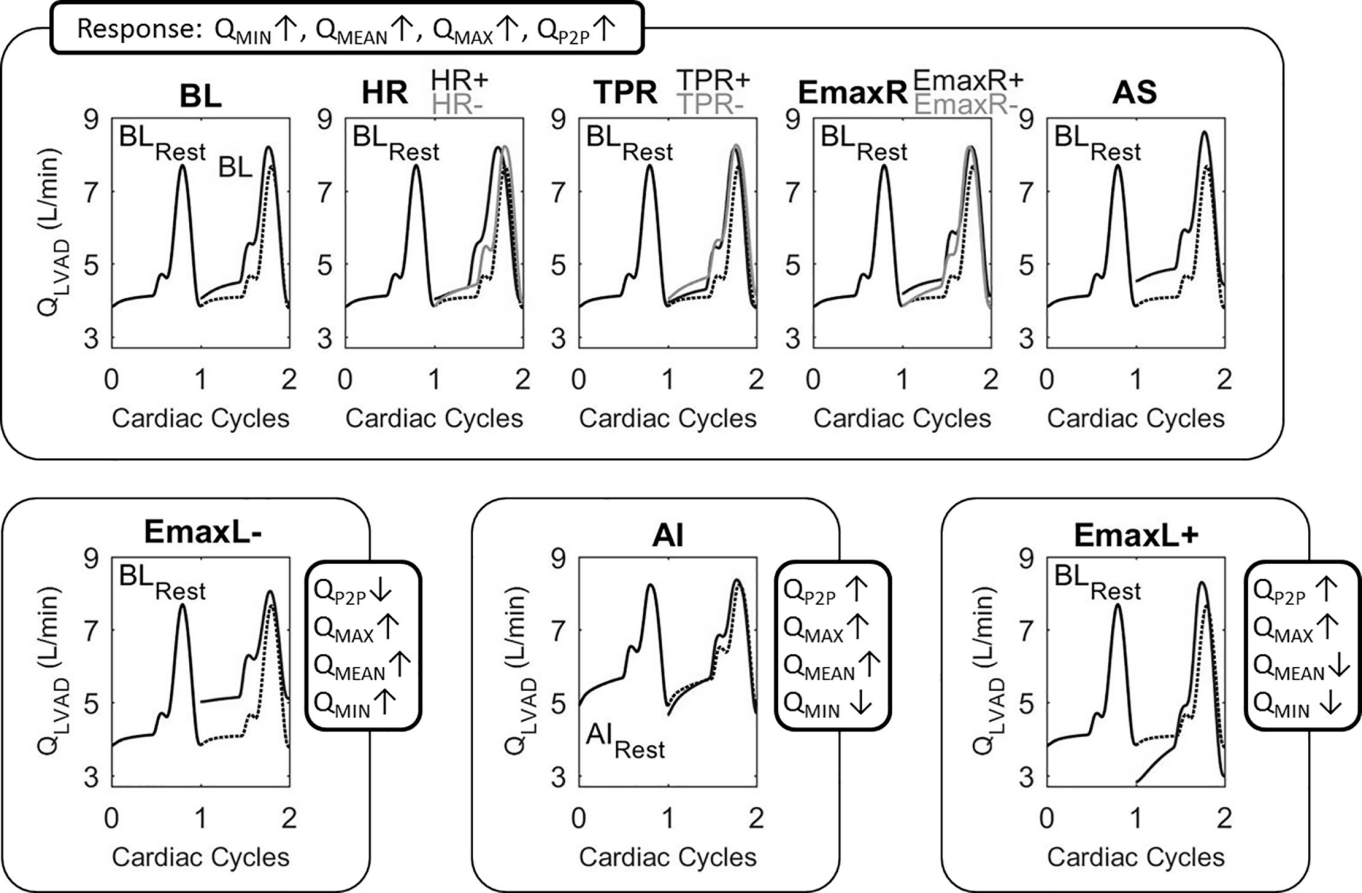
LVAD waveform responses from simulations. Time normalized pump flow waveforms for simulations resulting in the LVAD waveform parameter responses observed in patients. Heartrate for BL_REST_ was 75 bpm, for AI_REST_ 80 bpm, for HR- 90 bpm, for HR+ 136 bpm and ranging from 112 to 114 bpm for all other exercise simulations. Abbreviations and overview of simulations are shown in Table 1.

Simulations refer to the exercise response of the average LVAD patient from literature (BL) and to the additional responses summarized in Table 1. Simulated LVAD flow was analyzed and classified with the same method used for clinical data. In the simulations the LVAD waveform parameter responses already identified from patient data occurred (see Fig 5).

**Fig 6:**
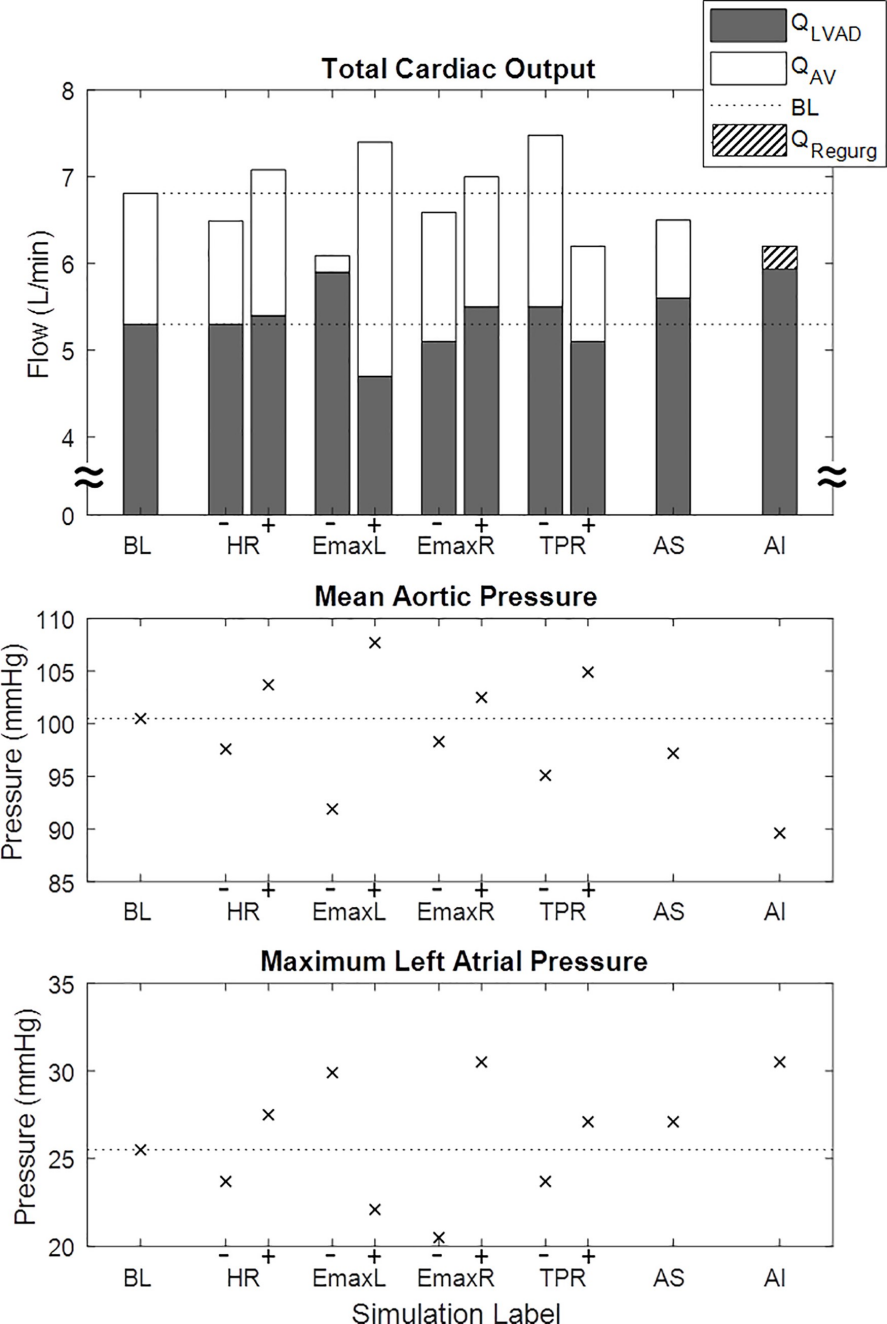
Hemodynamics at exercise from simulations. Total cardiac output and its repartition between the LV and LVAD are shown together with mean aortic pressure and maximum left atrial pressure for each simulation at exercise. Abbreviations and overview of simulations are shown in Table 1. (QRegurg: aortic valve regurgitant flow due to valve insufficiency; Q_LVAD_ equals Q_MEAN_; dotted line indicates levels for BL).

Response type 1 (Q_MIN_↑, Q_MEAN_↑, Q_MAX_↑, Q_P2P_↑) was observed in most of the simulations: BL, HR-, HR+, EmaxR-, EmaxR+, TPR+, TPR-, and AS. Response type 2 (ΔQ_MIN_↑ and ΔQ_P2P_↓) was observed for the simulation EmaxL-. The ΔQ_MIN_↓ and ΔQ_P2P_↑ from Response type 3 was observed for the simulations EmaxL+ and AI. A negative ΔQ_MEAN_ of -0.1 L/min was observed for the simulation EmaxL+, corresponding to an exceptionally good left ventricular contractility. Furthermore for each simulation at exercise hemodynamics such as total cardiac output and its repartition, preload and afterload surrogates are shown in Fig 6.

## Discussion

This study shows diverse responses in hemodynamic LVAD flow during exercise. The currently held assumption on the response of the LVAD to exercise predicts only one of the different responses observed (Response type 1), while this study also shows for the first time different LVAD waveform responses that shed light on the remaining cardiac function and overall on the patient-LVAD interaction. These observations were possible because of a unique monitoring device that records high resolution pump flow waveforms, which is not an integral part of the currently clinical available device. To understand the mechanisms underlying the hemodynamic LVAD responses observed in patients, numerical cardiopulmonary simulations were used.

### LVAD waveform responses

The currently held assumption posits, that LVAD flow rate and waveform parameters increase with preload (as a surrogate for physical activity or exercise) [30–32]. To better describe the LVAD interactions with the cardiovascular system during exercise, we introduced a classification strategy based on relevant and easily observable LVAD waveform parameters: Q_MIN_, Q_MEAN_, Q_MAX_ and Q_P2P_. The analysis we conducted on LVAD waveforms at exercise, revealed that also a decrease in Q_P2P_ or a decrease in Q_MIN_ with exercise was observed in some patients.

Response type 1 (ΔQ_MIN_↑, ΔQ_MEAN_↑, ΔQ_MAX_↑, ΔQ_P2P_↑) supports the assumed LVAD response to exercise and was found in the majority of exercise tests performed by patients and in most results from the simulations. This response resulted from patients with an open AV at peak exercise, as well as patients remaining in full-support. Similar responses were replicated by BL (the average patient simulation) as well as all simulations with superimposed parameters (HR±, EmaxR±, TPR± and AS) except for EmaxL± and AI.

Response type 2 (ΔQ_P2P_↓) and response type 3 (ΔQ_MIN_↓) were found in a minority of exercise tests, all in patients with AV opening at peak exercise indicating associations of the LV remaining contractile reserve. Indeed the results of the simulations support the importance of the output through the AV to differentiate between the two waveform responses to exercise. The decline in Q_P2P_ occurred in the simulations with a poor LV (EmaxL-), whereas the decline in Q_MIN_ resulted from the simulations with a higher LV inotropic response or in presence of AI.

### LVAD flow parameters

The mechanisms responsible for the change in beat-to-beat LVAD parameters were hypothesized to correspond to the hemodynamics from the systematic simulations. The cardiorespiratory simulator provided helpful insights in underlying mechanisms difficult to analyze in patients due the complexity with highly individual cardiac and peripheral responses to exercise, confounding factors and the sparse availability of hemodynamic assessments in patients during exercise tests.

#### Q_MAX_

The increase in Q_MAX_ observed at exercise, is due to an increase in the peak LV pressure. This might be due to a residual inotropic response of the LV and/or to an increased venous return that would generate a higher systolic LV pressure according to the Frank–Starling mechanism. Similar ΔQ_MAX_ with +0.5 L/min occurred for BL, HR± and EmaxR± (* further discussion on right heart function below). A ΔQ_MAX_ increase of +0.4 L/min was observed with exercise for the simulations EmaxL- and TPR+ and of +0.6 L/min for EmaxL+ and TPR-. Reduced ΔQ_MAX_ of +0.2 L/min occurred for AI. Increased ΔQ_MAX_ of +0.9 L/min occurred for AS, due to the high systolic pressure gradient across the AV in presence of AS.

#### Q_MIN_

The LVAD parameter during diastole (Q_MIN_) is related to the systolic arterial pressure as well as preload (filling ventricular pressure). During exercise, these two variables are affected by the increase in cardiac output, peripheral vasodilation and increase in venous return. In the majority of the simulations (BL, HR±, TPR±, EmaxR+ and AS) ΔQ_MIN_ increased (range +0.1 to 0.6 L/min) with exercise. This behavior results from a higher increase in preload compared to afterload at timepoint of Q_MIN_ in the cardiac cycle (observable as t2 in Fig 3) [33]. Furthermore the modulation of LV contractility produced the ranges in simulated ΔQ_MIN_ responses with –1.0 L/min for EmaxL+ and +1.3 L/min for EmaxL-, mostly mediated by the effect that an increased contractility has on the systolic arterial pressure.

#### Q_P2P_

Depending on the rate of change from rest to exercise in Q_MIN_ and Q_MAX_ the resulting ΔQ_P2P_ increases, decreases or might remain constant. A decrease in Q_P2P_ resulted only in the simulation EmaxL-, due to the high ΔQ_MIN_ corresponding to the high preload levels.

#### Q_MEAN_

The parameter Q_MEAN_ is calculated over the whole cardiac cycle and therefore an indicator for overall hemodynamic changes rather than particular cardiovascular impairments. Generally, due to the instantaneous increase in preload with dynamic exercises a sudden increase of Q_MEAN_ can be observed [4,32,34–36]. However Q_MEAN_ can increase to a different extent according to the residual ventricular contractility, LVAD speed setting as well as other parameters. The simulation with an exceptionally high LV contractility showed even a minor reduction of Q_MEAN_ during exercise compared to rest.

The simulations EmaxR± indicate that the LVAD response classification with exercise, used in this study, might not be sensitive to stratify for differences in EmaxR. Indeed the clinical observations also do not show clear differences in patients with different RV function. However hemodynamic differences according to EmaxR could be noticed: For the exercise simulation EmaxR- a reduction in left atrial pressure, aortic pressure, total cardiac output and Q_MEAN_ occurred, compared to EmaxR+ (see Fig 6). These hemodynamic differences mainly affect the diastolic part of the LVAD flow waveform (see Fig 5). (Additional information on cardiac output for the simulations are published in Gross et al. [37]).

### Clinical interpretation and relevance

Constant speed LVAD devices lack adequate adaption of pump output to exercise levels resulting in the hypoperfusion of patients during stress [3,34]. The observed differences in Q_MEAN_ responses from rest to exercise in patients and simulations underline that the support of the device in sustaining cardiac output is different among patients and can be influenced by several cardiovascular parameters [37]. Clinical observations in LVAD patients during exercise with increased as well as decreased LVAD speeds have demonstrated effects on exercise hemodynamics in patient subgroups, but not in the whole study cohorts [38,39]. The diverse characteristics of LVAD patients’ responses during exercise necessitate patient stratification. For this purpose, clinically validated beat-to-beat hemodynamic parameters can be helpful and easily obtained from continuous LVAD monitoring such as used in this study [6,8]. For exercise studies comparing baseline LVAD speed to increased speeds we propose the following patient stratification based on the baseline speed observations (A-D):

- AV closed at peak exercise (Response type 1 with AV closed): Due to the inability of the LV to increase total cardiac output, LVAD speed increase most probably will increase total cardiac output and increase unloading, in case of a non-failing RV.
- AV open at peak exercise (Response type 2): Patients may tolerate the highest LVAD speeds at exercise due to the impairment of the LV and high pulmonary pressures. These patients may benefit the most from LVAD speed increase during exercise in terms of total cardiac output.
- AV open at peak exercise (Response type 1 with AV open): This category will include the most patients and benefits with speed increase will most probably result in outcomes similar to previous studies [23–27,36].
- AV open at peak exercise (Response type 3): These patients may experience minor benefits of a speed increase during exercise due to a remaining LV contractility and its ability to increase total cardiac output. LVAD speed increase during exercise in this category could result in the redistribution of cardiac output between the LVAD and what it is ejected towards the aortic valve without the degree of increase of overall flow as it might be observed in others [37].

Furthermore, the proposed stratification could be helpful for physiological LVAD speed control algorithms to set the appropriate support mode to provide adequate support during exercise. Up to now most LVAD speed control algorithms are studied in-silico, ex-vivo or in-vivo in animals without heart failure. In-silico or ex-vivo studies could be carried out to reproduce the diverse responses found in patients in order to improve algorithms based on clinically observed evidence. For this purpose, the individual responses are available in the online data of this study (S1 Table). Characterizing the individual patient’s cardiac, peripheral and LVAD response during exercise could have implications in diagnostics and clinical management of LVAD patients and help to drive technological advancements of LVADs towards “smart pumping”. The potential stratification proposed in this section could be the foundation for further work, following clinical validation. This study comprises useful methods and reports possible mechanisms for the different responses helpful to design and carry out such clinical validation. The intra-individual differences in responses observed in two patientns emphasize the need for repeated tests performed, together with the proper clinical assessments for e.g.: volume status, right and left ventricular function. Further work may include more parameters for response stratification as shown in Table 3.

### Limitations

For the patient analysis only 14 patients received the high-resolution LVAD data recorder and performed maximum bicycle exercise tests. Cardiopulmonary exercise tests and bicycle stress-echocardiography were combined, nevertheless due to the occurrences of all response types in both bicycle exercise test modalities no systematic error was introduced. Furthermore, variables as cardiac output and invasive pressure measurements were not available during exercise tests. Some of the occasionally measured non-invasive blood pressures were unreliable, due to the low arterial pulse pressure with an LVAD, and therefore not analyzed in this study. Only the HVAD pump was studied however we would not expect major differences at least for other centrifugal pumps (Heartmate 3) Despite these limitations, this study allowed the observation of hemodynamic responses to exercise not yet reported in the literature, which were corroborated by extensive numerical simulations, in which variables as cardiac output or arterial pressure behaved as reported in clinical observations.

In this study systematic simulations based on the average LVAD patient data from literature were performed, and the effect of single parameter changes investigated. This is different in real patients as multiple concomitant conditions might be present simultaneously. Nevertheless this approach can show that specific changes in parameter from the average patient (e.g. lower LV contractility) might explain some of the peculiar exercise responses observed (e.g. decrease in flow pulsatility). Worth to mention is that the simulation with a reduced (-20%) right ventricular contractility labeled as EmaxR-, cannot be interpreted as or correlated to hemodynamics resulting with a failing right ventricle.

## Conclusion

Diverse responses in pump flow patterns were observed in LVAD patients performing bicycle exercise stress tests reflecting differences in the underlying remaining cardiac condition as one of the major determinants for the responses. In patients with poorer contractility exercise-responsive LVAD control might bring additional benefits in terms of adequate support of the failing left ventricle, the patient’s exercise capacity and resulting quality of life.

## Supporting information

S1 Table Hemodynamic LVAD responses for each exercise test. Patients’ LVAD parameters at baseline and peak exercise and response type classification with constant LVAD speeds.
(XLS)

## Abbrevations

AV: aortic valve
AI: Aortic Insufficiency
AS: Aortic Stenosis
EmaxL/EmaxR: left/right ventricular contractility
HR: heartrate
LVAD: left ventricular assist device
TPR: total peripheral resistance
Q_MAX_: pump flow maximum
Q_MIN_: pump flow minimum
Q_MEAN_: pump flow mean
Q_P2P_: pump flow pulsatility
